# Splenectomy

**Published:** 1890-05-03

**Authors:** 


### SPLENECTOMY.

Last year two cases of excision of the spleen were reported in the English medical journals; one performed on a Hindu by Mr. Hatch, which ended fatally from hæmorrhage and collapse, the other by Sir Spencer Wells, with ultimate recovery after numerous complications.

But an operation performed last winter by Dr. Gussenbauer is specially deserving of notice not only from the complete success attending it, but on account of the novelty of his procedure. He, for the first time, used the thermocautery, thus arresting the hæmorrhage as the section was carried slowly through the transverse diameter corresponding to the middle of the organ. In two places only was it necessary to employ the artery forceps and torsion. Recovery was rapid, uninterrupted, and complete.

